# An exploratory Mendelian randomization study on the genetically predicted effects of circulating blood cells on osteoarthritis risk

**DOI:** 10.1016/j.clinsp.2026.100959

**Published:** 2026-04-17

**Authors:** Lei Zhou, Jialin Jia, Xingren Chen, Tong Chen

**Affiliations:** Department of Orthopedics, Beijing Chaoyang Hospital, Capital Medical University, Beijing, China

**Keywords:** Mendelian randomization, Circulating blood cells, Osteoarthritis, GWAS

## Abstract

**Background:**

The causal relationship between circulating blood cells and Osteoarthritis (OA) remains unclear. This study aimed to explore this potential relationship using a two-sample Mendelian Randomization (MR) approach.

**Methods:**

Data from large-scale GWAS (Blood Cell Consortium and GWAS Catalog) were analyzed using two-sample MR methods, with Inverse Variance Weighting as the primary approach. A Bonferroni correction was applied for multiple testing for the two primary traits. Robustness was assessed through comprehensive sensitivity analyses for heterogeneity and pleiotropy.

**Results:**

After Bonferroni correction, Genetically Predicted Hemoglobin (HGB) showed a weak genetic association consistent with a potential causal pathway for OA risk (OR = 1.004, 95% CI 1.001–1.007, p = 0.018). The association for Neutrophil (NEU) count was not statistically significant. While sensitivity analyses did not detect directional pleiotropy, significant heterogeneity was observed for the genetic instruments.

**Conclusion:**

This exploratory study provides preliminary genetic evidence for a statistically significant but clinically negligible MR estimate of hemoglobin in OA pathogenesis. The clinical and biological significance of this minuscule effect remains uncertain. The findings should be interpreted as hypothesis-generating, highlighting the need for further research rather than establishing a definitive causal link.

## Highlights

• Genetically predicted hemoglobin levels show a weak potential association with increased osteoarthritis risk.
• Neutrophil count is not significantly associated with osteoarthritis risk after correction for multiple testing.
• The effect size for hemoglobin is clinically negligible, suggesting an exploratory rather than definitive causal link.
• Significant heterogeneity and potential pleiotropy suggest these findings should be interpreted with caution.

## Introduction

Osteoarthritis (OA) is a prevalent musculoskeletal disorder and a leading cause of disability worldwide.^1^ It is characterized by the degeneration of articular cartilage and underlying bone, resulting in pain, stiffness, and functional impairment.^2^ OA imposes a substantial burden globally, impacting both the physiological and psychological health of patients and incurring significant healthcare costs.^3^ Risk factors for OA include genetic predisposition, age, obesity, and trauma.^4^, ^5^, ^6^, ^7^ However, its etiology remains incompletely understood. Therefore, identifying potential and modifiable risk factors is crucial for elucidating the pathogenesis of OA and is essential for reducing its incidence rates.

In OA research, circulating blood cells and systemic inflammatory mediators play a critical role in the disease's pathogenesis. Numerous studies have demonstrated that various immune cells, including leukocytes (particularly monocytes), macrophages, T-cells, and platelets, are integral to the chronic inflammatory response, bone resorption, and cartilage degradation associated with OA.^8^^,^^9^ Monocytes exhibit altered inflammatory responses in OA patients,^8^ while macrophages mediate the production of cytokines and Matrix Metalloproteinases (MMPs) that drive joint degeneration.^10^ Furthermore, specific T-cell subsets, such as pro-inflammatory Th17 and Th9 cells, are elevated in OA patients and contribute to cartilage destruction through T-cell-secreted-cytokine-mediated pathways.^11^, ^12^, ^13^ These studies underscore the complex involvement of immune cells in the chronic inflammation seen in OA.

While current studies provide important insights, the present study specifically focuses on widely measured and standardized erythrocyte and leukocyte parameters (hemoglobin and neutrophils). This focus is rationalized because these parameters have strong genetic instruments available in large-scale GWAS, represent fundamental physiological systems (oxygen transport and innate immunity), and have been implicated in systemic inflammation, a known contributor to OA. It is acknowledged that previous Mendelian randomization studies have investigated the role of iron status (which influences hemoglobin).^14^^,^^15^ and broader categories of immune cells in OA.^9^ This study aims to build upon this by examining these specific blood cell traits. Most existing research relies on observational designs, which are susceptible to confounding factors and reverse causality biases, leading to inconsistent conclusions.^13^ For example, the role of platelets in OA is complex; they are implicated in OA inflammation but are also used as a basis for therapeutic strategies.^16^ These inconsistencies highlight the need for new research approaches to clarify the complex relationship between circulating blood cells and OA.

To overcome these limitations, Mendelian Randomization (MR) analysis, a causal inference method, offers a powerful tool. MR uses genetic variations as Instrumental Variables (IVs) to mitigate bias from confounding factors and reverse causality.^17^^,^^18^ It has been successfully applied to investigate various health outcomes.^19^^,^^20^ By using MR, the authors can obtain more reliable evidence on the potential causal role of exposures like circulating blood cells in OA. However, MR relies on key assumptions that must be carefully evaluated and interpreted within its inherent limitations. This study was conducted and reported following the principles outlined in the STROBE-MR guidelines.^21^

In this study, the authors employ MR to evaluate the potential causal relationship between specific circulating blood cell traits and OA. The authors aim to provide additional insights into the role of these blood cells in the pathogenesis of OA, thereby contributing to the ongoing effort to identify novel prevention and treatment strategies.

## Materials and methods

### Research design

**Fig. 1:**
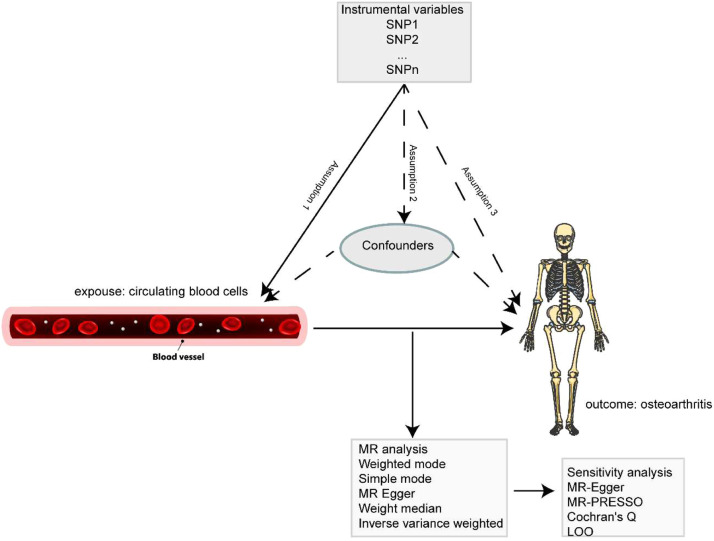
Flowchart of a MR study.

This study utilizes a two-sample MR approach to assess the causal relationship between circulating blood cells and OA. MR studies must satisfy three essential assumptions: 1) Genetic variants should be significantly associated with the exposure (relevance assumption); 2) The genetic variants used as instruments should be independent of any potential confounders (independence assumption); 3) The genetic variants should influence the outcome solely through their effect on the exposure (exclusion restriction assumption). Fig. 1 depicts the overall MR study process.

### Data sources

Data for this study were sourced from publicly available GWAS databases. Genetic variants related to circulating blood cells were obtained from the Blood Cell Consortium (BCX), encompassing data on red blood cells, white blood cells, and platelets.^22^ The authors analyzed data from European populations, with further details available at Chen et al., Cell 2020.^22^ Genetic data related to osteoarthritis were retrieved from the GWAS Catalog (Dataset: ebi-a-GCST90038686), which includes 484,598 participants (39,515 cases and 445,083 controls). Specific research data can be found on the website. It is important to note that all GWAS data used are based on European populations, which limits the generalizability of these findings.

### Instrumental variables (IVs) selection

To ensure the validity of the MR analysis, the authors implemented a rigorous SNP selection process. First, the authors extracted SNPs related to blood cell types from the GWAS data at a genome-wide significance threshold (p < 5 × 10^–8^). Next, to address Linkage Disequilibrium (LD), the authors employed the “clump_data” function with parameters set to κb = 10,000 and *r*^2^ = 0.001. Furthermore, the authors validated the selected SNPs against potential confounding factors using the Phenoscanner database (www.phenoscanner.medschl.cam.ac.uk).^23^ SNPs found to be strongly associated with known OA risk factors independent of the blood cell trait were considered for exclusion. During data coordination, the authors excluded SNPs with inconsistent allele frequencies or those exhibiting palindromic structures. Finally, the authors calculated the F-statistic for each SNP to assess instrument strength, retaining only those with an F-statistic greater than 10 to minimize weak instrument bias.

### MR analysis

The authors employed multiple MR analysis methods, including Inverse Variance Weighting (IVW), MR-Egger regression, Weighted Median, Simple Model, and Weighted Model. IVW was selected as the primary analysis method due to its high statistical power, assuming no unbalanced horizontal pleiotropy. To complement the IVW analysis, the authors utilized MR-Egger regression to assess directional horizontal pleiotropy, and the Weighted Median and other models as sensitivity analyses to test the consistency of the results under different assumptions. In this study, the authors focused on two primary blood cell traits (HGB and NEU) to represent the erythroid and myeloid lineages, respectively. Consequently, the authors applied a Bonferroni correction for these two primary tests, setting the significance threshold at p < 0.025 (0.05/2).

### Sensitivity analysis

To validate the robustness of the MR analysis, several sensitivity analyses were conducted. Cochran's *Q*-test evaluated heterogeneity among SNPs. The MR-Egger regression intercept was used to examine directional horizontal pleiotropy. The MR-PRESSO method was used to identify and correct for horizontal pleiotropic outliers. Additionally, the authors applied the Leave-One-Out (LOO) analysis to check whether any individual SNP disproportionately influenced the causal estimates. Finally, the authors utilized Steiger filtering to confirm the causal direction between the exposure and the outcome. All analyses were performed using R software (version 4.1.0) with the “TwoSampleMR” package.

## Results

### Selection of IVs

The authors implemented a rigorous selection process for genetic IVs (p < 5 × 10^–8^). For the HGB analysis, 454 SNPs were used, and for the NEU analysis, 415 SNPs were used. Overall, F-values for the selected instruments ranged from 29.76 to 6218.95, and the variance explained (R^2^) ranged from 0.01% to 1.27%. The mean F-statistic was 105.4 for HGB and 98.7 for NEU, suggesting a robust instrument strength overall. Summary information for the IVs is available in Supplementary Table 1.

### MR analysis

**Fig. 2:**
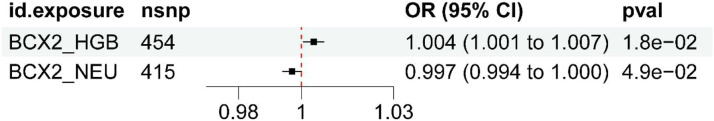
A forest plot showing the causal relationships between HGB, NEU, and osteoarthritis risk from the primary IVW analysis. The association for HGB is statistically significant after Bonferroni correction (p < 0.025), while the association for NEU is not.

**Fig. 3:**
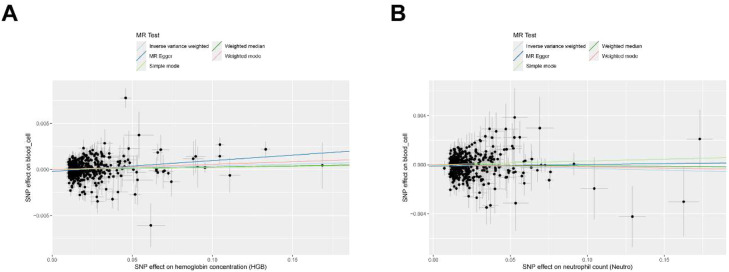
Scatter plot of circulating blood cells on the risk of osteoarthritis. (A) HGB; (B) NEU.

The two-sample MR analysis results are presented in Supplementary Table 2. After applying the Bonferroni correction (significance threshold p < 0.025), a genetically predicted increase in Hemoglobin (HGB) levels was causally linked to a slightly increased risk of OA (OR = 1.004, 95% CI 1.001–1.007, p = 0.018). The observed inverse association for Neutrophil (NEU) levels was not statistically significant after correction (OR = 0.997, 95% CI 0.994–1.000, p = 0.049, Bonferroni-corrected p > 0.025) (Fig. 2). The directional consistency across the other four MR methods (MR Egger, Weighted median, Simple mode, Weighted mode) provided some support for the findings, though statistical significance varied across methods (Fig. 3, Supplementary Fig. 1).

### Sensitivity analysis

**Fig. 4:**
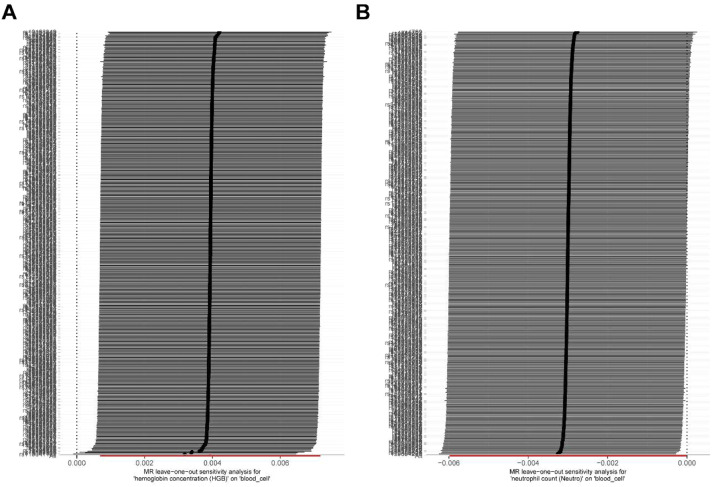
LOO analysis of circulating blood cells on the risk of osteoarthritis. (A) HGB; (B) NEU.

**Fig. 5:**
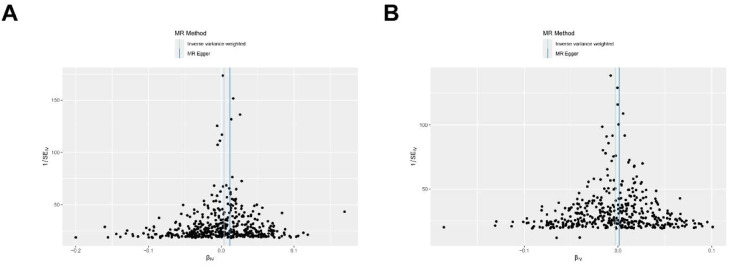
Circulating blood cells funnel plot for Mendelian randomization analysis. (A) HGB; (B) NEU.

To validate the reliability of the MR analyses, the authors assessed both pleiotropy and heterogeneity. The MR-Egger intercept did not indicate significant directional horizontal pleiotropic effects for HGB (p-intercept = 0.052) or NEU (p-intercept = 0.107) (Table 1). Similarly, the MR-PRESSO global test found no evidence of significant outlier-driven pleiotropy (Table 2). However, the Cochran's *Q*-statistic from the IVW method showed significant heterogeneity for both HGB (*Q* p-value < 0.001) and NEU (*Q* p-value < 0.001) (Table 3). This indicates variability in the SNP-specific estimates. The Leave-One-Out (LOO) analysis revealed that the exclusion of any individual SNP did not substantially alter the causal estimates, confirming the stability of the results (Fig. 4). The Steiger filtering test confirmed the assumed causal direction from blood cell traits to OA (Table 4). Funnel plots for HGB and NEU showed some asymmetry, consistent with the heterogeneity detected by the Cochran's *Q*-test (Fig. 5).

**Table 1:** Detection of directional pleiotropy using MR-Egger regression.

Exposure	Outcome	Egger Intercept	SE	p-value
Hemoglobin concentration (HGB)	Osteoarthritis	−0.000217	0.000111	0.052
Neutrophil count (NEU)	Osteoarthritis	−0.000118	0.000073	0.107

**Table 2:** Global heterogeneity test using Mendelian Randomization (MR) of Circulating Blood Cells and Osteoarthritis (MR-PRESSO).

MR Analysis	Causal Estimate	SD	T-stat	p-value	ID	RSSobs	Global.Test. p-value
Raw	0.003137788	0.001619943	1.936975031	0.053341006	BCX2_HGB	731.2481397	0.00205761316872428
Outlier-corrected	0.002674821	0.001497021	1.786762353	0.074621662	BCX2_HGB	NA	NA
Raw	−0.002524781	0.00153499	−1.644819124	0.100737913	BCX2_NEU	586.8553111	0.00226757369614512
Outlier-corrected	−0.002888369	0.001470779	−1.963836249	0.050197462	BCX2_NEU	NA	NA

**Table 3:** Detection of heterogeneity using the Cochran *Q*-test of IVW method.

id.exposure	id.outcome	Outcome	Exposure	Method	*Q*	*Q*_df	*Q*_pval
BCX2_HGB	ebi-a-GCST90038686	Osteoarthritis	Hemoglobin concentration (HGB)	Inverse variance weighted	695.89	453	1.48E-12
BCX2_NEU	ebi-a-GCST90038686	Osteoarthritis	Neutrophil count (Neutro)	Inverse variance weighted	528.96	414	0.000108549

**Table 4:** Detection of causal direction using the Steiger test.

id.exposure	id.outcome	Exposure	Outcome	snp_r2.exposure	snp_r2.outcome	correct_causal_direction	steiger_pval
BCX2_HGB	ebi-a-GCST90038686	Hemoglobin concentration (HGB)	Osteoarthritis	8.49E-05	2.68E-08	TRUE	4.22E-06
BCX2_NEU	ebi-a-GCST90038686	Neutrophil count (Neutro)	Osteoarthritis	5.69E-05	1.11E-08	TRUE	0.00020683

## Discussion

This study employed Mendelian randomization to explore the causal effects of circulating blood cell traits on OA risk in European populations. This analysis identified a statistically significant but clinically minuscule causal association between genetically predicted HGB levels and an increased risk of OA. In contrast, the association for NEU levels did not withstand correction for multiple testing. The odds ratio for HGB (OR=1.004) indicates a mere 0.4% increase in OA risk per unit increase in genetically predicted HGB, an effect size dwarfed by established risk factors like obesity.^24^ and unlikely to be meaningful for individual risk prediction. It is important to interpret these findings in the context of existing literature; the link between iron status (related to HGB) and OA has been explored in previous MR studies.^14^^,^^15^ and this finding for HGB is consistent with reports of higher iron levels associated with a small increased OA risk. The present study thus adds to this body of evidence but does not represent a transformative discovery.

The finding that higher HGB is a risk factor for OA is biologically plausible. Hemoglobin, the key oxygen-transporting protein in red blood cells.^25^ is intrinsically linked to iron metabolism. Elevated systemic iron levels, potentially reflected by higher HGB, have been causally associated with increased OA risk in other MR studies.^14^^,^^15^ The proposed mechanism involves iron-induced oxidative stress, which can promote chondrocyte senescence and cartilage degradation.^26^^,^^27^ Excess iron can also lead to ferroptosis in chondrocytes, contributing to joint damage.^28^ Previous research also found that elevated glycated Hemoglobin (HbA1c) levels are causally associated with an increased risk of knee OA.^29^ Therefore, these finding aligns with established biological pathways linking iron overload and metabolic dysfunction to OA pathogenesis. Given the tiny effect size, it is also possible that HGB acts as a proxy for other correlated factors such as altitude exposure, cardiorespiratory fitness, or smoking status, rather than exerting a direct biological effect.^30^^,^^31^

The initial, uncorrected finding of a protective association between higher neutrophil levels and OA risk (p = 0.049) was not robust to multiple testing correction. This result is also biologically counterintuitive and contradicts a substantial body of literature. Neutrophils are key mediators of acute inflammation and are implicated in the chronic inflammatory processes of OA. They contribute to cartilage degradation through the release of enzymes like neutrophil elastase and the formation of Neutrophil Extracellular Traps (NETs) within the joint.^32^^,^^33^ The speculative hypothesis that systemic neutrophil levels might be protective via anti-inflammatory Extracellular Vesicles (EVs).^34^ is not sufficient to overturn this well-established pro-inflammatory paradigm in OA. A more parsimonious explanation for the initial borderline association is that it represents a false positive (Type I error), residual confounding, or complex horizontal pleiotropy, where the genetic variants for neutrophil count influence OA through pathways unrelated to neutrophil function itself.

### Limitations and methodological considerations

This study has several important limitations. First, these findings of statistically significant but minuscule genetic effects must be interpreted with extreme caution. The clinical utility of these associations for risk prediction or therapeutic development is likely negligible in their current form. Their primary value is in generating hypotheses about underlying biological pathways.

Second, the significant heterogeneity detected by the Cochran's *Q*-test (p < 0.001 for both HGB and NEU) is a major concern. Such profound heterogeneity suggests that the genetic instruments are not acting with a consistent effect on the outcome, which challenges the validity of the primary IVW estimate. While the sensitivity analyses did not detect directional pleiotropy, the presence of widespread, balanced pleiotropy cannot be excluded and may be driving the observed heterogeneity. However, it is crucial to note that while the IVW method yielded a significant result, the MR-PRESSO outlier-corrected estimate (Table 2) did not reach statistical significance (p = 0.075), suggesting that the IVW result may be driven by horizontal pleiotropy or outliers. This discrepancy, combined with the extreme heterogeneity, indicates that the IVW estimate may not be a valid causal estimate and should be interpreted with extreme caution.

Third, the present study is based exclusively on data from individuals of European ancestry. This significantly limits the generalizability of these findings to other populations. Genetic architecture and gene-environment interactions can vary across different ancestries, and these results may not be representative of the global OA burden.^35^ Future replication in large, diverse cohorts is essential to validate these findings.

Fourth, while the authors attempted to address multiple comparisons by applying a Bonferroni correction, this exploratory study scanned multiple blood cell traits initially. The borderline significance of the main findings highlights the risk of false positives inherent in such an approach. Finally, MR studies assess the lifelong effect of genetically predicted exposure levels, which may not correspond to the effects of interventions that modify blood cell counts later in life. Although the mean F-statistics were high, the wide range indicates some instruments were weaker, which, combined with the tiny effect size, could introduce weak instrument bias. Finally, due to the widespread heterogeneity and likely pleiotropy, a formal colocalization analysis was not performed as the assumption of a single causal variant was likely violated.

In conclusion, this exploratory MR study provides weak evidence for a potential causal link between genetically elevated HGB and a very small increase in OA risk. The finding for neutrophils was not statistically significant after appropriate correction. Given the tiny effect size, unresolved heterogeneity, and other limitations, these findings should be considered preliminary and hypothesis-generating.

## Conclusion

This Mendelian Randomization study explored the genetically predicted effects of selected blood cell traits on osteoarthritis risk in European-ancestry individuals. This analysis found a statistically significant but minuscule genetic association between higher Hemoglobin (HGB) levels and increased OA risk, while the association for Neutrophil (NEU) counts was not significant after correction for multiple testing. The biological and clinical relevance of the HGB finding remains uncertain due to its negligible effect size. The study’s conclusions are constrained by significant heterogeneity and a lack of generalizability beyond European populations. Therefore, these results should be interpreted as exploratory and are insufficient for making any clinical inferences. Future research with more diverse datasets and functional studies is required to clarify the potential role, if any, of hematological parameters in OA pathogenesis.

## Data availability

This study analyzed publicly available datasets. Genetic association data for circulating blood cells were obtained from the Blood Cell Consortium (BCX) as described by Chen et al.^22^ Genetic association data for osteoarthritis were obtained from the GWAS Catalog (Dataset: ebi-a-GCST90038686; https://www.ebi.ac.uk/gwas/studies/GCST90038686). Original data sources are cited in the manuscript.

## Clinical trial number

Not applicable.

## Ethics approval and consent to participate

Ethical approval was not required for this study, as all data were sourced from publicly accessible summary-level GWAS databases. The original studies that generated these data obtained appropriate ethical approval and participant consent.

## Consent for publication

Not applicable.

## Authors’ contributions

Lei Zhou, Jialin Jia, Xingren Chen, and Tong Chen made substantial contributions to the conception and design of the study, searching the literature, and extracting and analyzing data. Lei Zhou wrote the manuscript; Tong Chen revised the manuscript; All authors approved the final version of the manuscript.

## Funding

No funding was received for this study.

## Declaration of competing interest

The authors declare no conflicts of interest.

## Appendix Supplementary materials

Image, application 1

Image, application 2
