# Supplementary material for: An exploratory Mendelian randomization study on the genetically predicted effects of circulating blood cells on osteoarthritis risk
Source: Clinics (Sao Paulo). 2026 Apr 17;81:100959. doi: 10.1016/j.clinsp.2026.100959 (PMC13098457; doi:10.1016/j.clinsp.2026.100959)

CLINICS-D-25-00845_Supplementary Material

**Supplementary Table S1** Instrumental variables used in MR analysis of the association between circulating blood cells and osteoarthritis

**Supplementary Table S2** Mendelian randomization approaches were employed to investigate the association between circulating blood cells and osteoarthritis

**Supplementary Figure 1** Five Mendelian randomization analyses were conducted to assess the causality between different exposures and outcome. (A) Preliminary MR analysis of the association between circulating blood cells and osteoarthritis. (B) Forest plot showing the results of five Mendelian randomization methods.


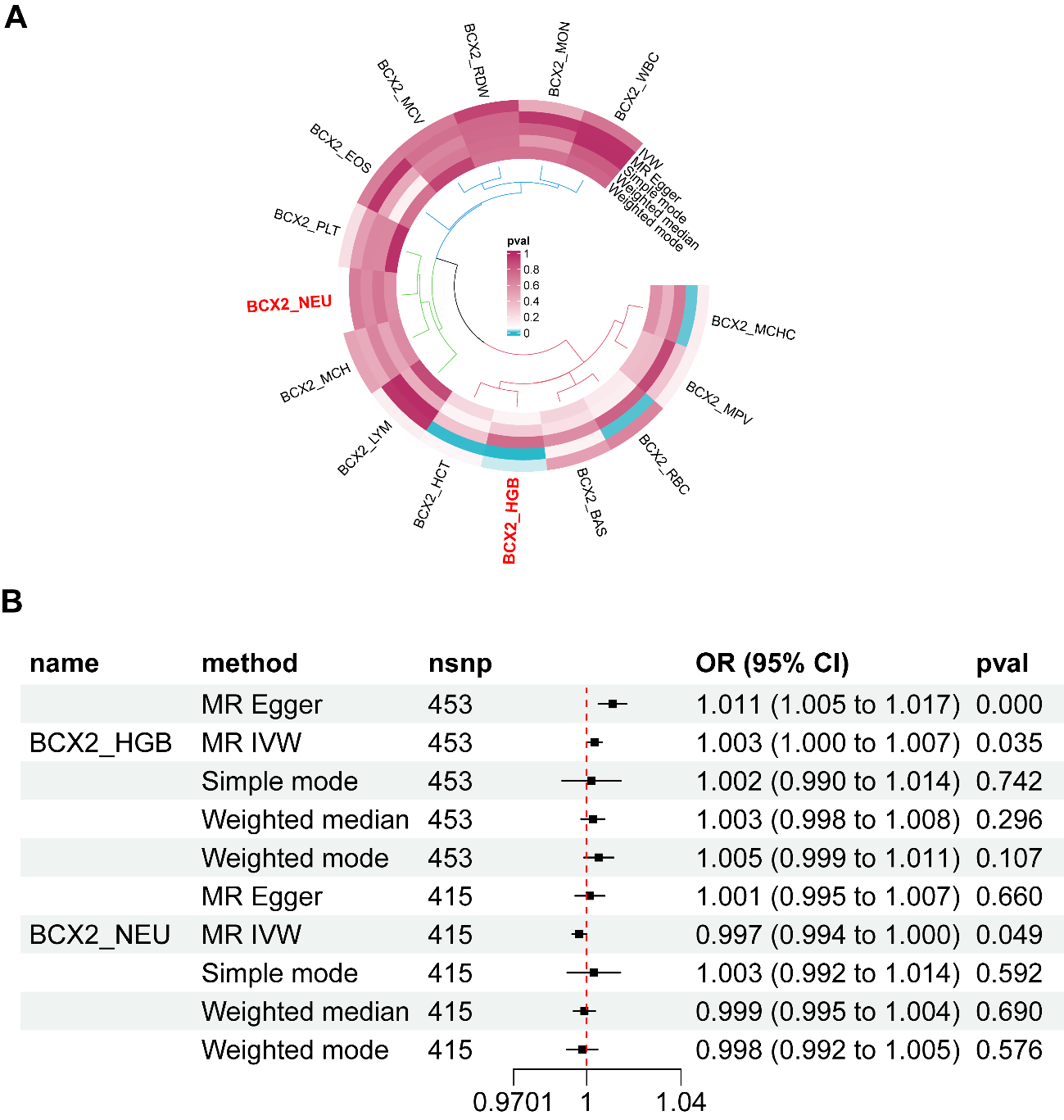

Supplement: Supplementary file 1 [file mmc1.docx]
